# A protocol for the identification and validation of novel genetic causes of kidney disease

**DOI:** 10.1186/s12882-015-0148-8

**Published:** 2015-09-15

**Authors:** Andrew Mallett, Chirag Patel, Barbara Maier, Julie McGaughran, Michael Gabbett, Minoru Takasato, Anne Cameron, Peter Trnka, Stephen I. Alexander, Gopala Rangan, Michel C. Tchan, Georgina Caruana, George John, Cathy Quinlan, Hugh J. McCarthy, Valentine Hyland, Wendy E. Hoy, Ernst Wolvetang, Ryan Taft, Cas Simons, Helen Healy, Melissa Little

**Affiliations:** 1 Kidney Health Service and Conjoint Kidney Research Laboratory, Royal Brisbane and Women’s Hospital, Brisbane, Australia; 2 Centre for Kidney Disease Research, Centre for Chronic Disease and CKD.QLD, School of Medicine, The University of Queensland, St Lucia, Australia; 3 Institute for Molecular Bioscience, The University of Queensland, St Lucia, Australia; 4 Genetic Health Queensland, Royal Brisbane and Women’s Hospital, Brisbane, Australia; 5 Murdoch Children’s Research Institute, Royal Children’s Hospital, Melbourne, Australia; 6 School of Medicine, Griffith University, Brisbane, Australia; 7 Queensland Child and Adolescent Renal Service, Lady Cilento Children’s Hospital, Brisbane, Australia; 8 Department of Nephrology, Children’s Hospital at Westmead, Sydney and Sydney Medical School, The University of Sydney, Sydney, Australia; 9 Department of Nephrology, Westmead Hospital, Sydney and Sydney Medical School, The University of Sydney, Sydney, Australia; 10 Department of Genetic Medicine, Westmead Hospital, Sydney and Sydney Medical School, The University of Sydney, Sydney, Australia; 11 Department of Anatomy and Developmental Biology, School of Biomedical Sciences, Monash University, Melbourne, Australia; 12 Department of Nephrology, Royal Children’s Hospital, Melbourne, Australia; 13 Molecular Genetics Laboratory, Pathology Queensland and Royal Brisbane and Women’s Hospital, Brisbane, Australia; 14 Australian Institute for Bioengineering and Nanotechnology, The University of Queensland, St Lucia, Australia; 15 Department of Paediatrics, University of Melbourne, Melbourne, Australia; 16 Kidney Health Service, Level 9, Ned Hanlon Building, Royal Brisbane and Women’s Hospital, Butterfield Street, Herston, Brisbane, Qld 4029 Australia

**Keywords:** Chronic kidney disease, Nephrology, Nephrogenetics, Genetic sequencing, Induced pluripotent stem cell

## Abstract

**Background:**

Genetic renal diseases (GRD) are a heterogeneous and incompletely understood group of disorders accounting for approximately 10 % of those diagnosed with kidney disease. The advent of Next Generation sequencing and new approaches to disease modelling may allow the identification and validation of novel genetic variants in patients with previously incompletely explained or understood GRD.

**Methods/Design:**

This study will recruit participants in families/trios from a multidisciplinary sub-specialty Renal Genetics Clinic where known genetic causes of GRD have been excluded or where genetic testing is not available. After informed patient consent, whole exome and/or genome sequencing will be performed with bioinformatics analysis undertaken using a customised variant assessment tool. A rigorous process for participant data management will be undertaken. Novel genetic findings will be validated using patient-derived induced pluripotent stem cells via differentiation to renal and relevant extra-renal tissue phenotypes *in vitro*. A process for managing the risk of incidental findings and the return of study results to participants has been developed.

**Discussion:**

This investigator-initiated approach brings together experts in nephrology, clinical and molecular genetics, pathology and developmental biology to discover and validate novel genetic causes for patients in Australia affected by GRD without a known genetic aetiology or pathobiology.

## Background

Renal disease is common with reported prevalence ranging from 10 to 16 % of Australian adults [1–3]. The impact of renal disease on the Australian community in terms of morbidity, mortality and health service requirements is significant [4, 5]. Renal disease is the most common cause of hospital admission, with more than 1,000,000 admissions per year, and the cumulative cost of treating current and new cases of end stage kidney disease 2009–2020 is estimated at AUD$11.3–12.3 billion [6]. Genetic renal disease (GRD) accounts for approximately 10 % of patients with renal disease [7, 8], with an additional proportion not as yet identified as being GRD or even as having yet developed renal disease. Up to half of presently identified GRD cases remain genetically unexplained.

The impact of GRD is well demonstrated by the most common and best understood form: Autosomal Dominant Polycystic Kidney Disease (ADPKD). ADPKD has a population prevalence of 1/400-800 and is the most common potentially lethal genetic disease in humans [9]. In Australia and New Zealand, ADPKD is the primary renal diagnosis in 6 % of the 12,968 prevalent dialysis patients and the fourth most common cause for dialysis commencement [10, 11]. Effective medical therapies for this condition are on the cusp of clinical translation after several decades of research, which initially elucidated the genetic aetiology of ADPKD, then explained its pathogenesis, and finally enabled translational applications via clinical therapeutic trials [12]. ADPKD, however, represents less than half of the estimated cases of GRD with many other renal diseases being unidentified and incompletely understood. Studies aiming at the detailed phenotypic description and explanation of these other and often very rare forms of GRD, are needed.

Enormous progress has been achieved in the field of molecular genetics and genomics since the discovery of deoxyribose nucleic acid (DNA) in 1953 [13]. The emergence of massively parallel sequencing (MPS) from approximately 2005 [14] has arguably been the next greatest advance since the development of Sanger sequencing in the late 1970s [15, 16]. Next generation sequencing (NGS) collectively describes these recently-developed genetic technologies that enable rapid and cost effective sequencing of large amounts of DNA. While sequencing of the first human genome took over 10 years and required an international collaboration at the approximate cost of USD $3 billion [17], NGS now allows a human genome to be sequenced in days at the cost of only $1–2,000 [18]. Whole exome sequencing (WES) is a variation on whole genome sequencing (WGS) that allows for targeted sequencing of only the approximately 1.5 % of the human genome that contains protein-coding genes, reducing costs even further [19].

NGS facilitates rapid identification of disease-causing genetic variants in patients with a suspected Mendelian disorder. This utility was first proven by the discovery of the gene involved in causing Freeman-Sheldon syndrome [20]. With reduced cost and time, WES has become the preferred initial method for disease gene identification in small pedigrees, replacing previous methods, such as linkage analysis and association studies. By 2012, more than 100 causative genes had been identified by means of WES [14]. WES is now able to elucidate the causal mutations in nearly 50 % of patients thought to have a genetic condition [21–23]. This diagnostic yield is further optimised when family-based studies such as the trio/family-based approach are employed [24]. In nephrology, NGS has identified disease-causing mutations responsible for atypical haemolytic uremic syndrome [25, 26], steroid-resistant nephrotic syndrome [27–29] and nephronophthisis [30–39]. However, the resolution within which any base change can be identified using this technique results in the identification of large numbers of variations, not all of which are pathogenic. Hence, it is now more critical than ever to validate the pathogenicity of any novel genetic variant. This may include appropriate Mendelian segregation and genotype/phenotype correlation via some form of biological validation.

With the advent of reprogramming of adult cells to a pluripotent state, termed induced pluripotent stem cells (iPSC), clinicians now have an opportunity to differentiate cells from the patient in question into relevant tissue types and *in vitro* [40]. Patient-specific iPSC differentiation allows for more effective modelling of the patient’s own disease [41–44]. For renal disease, this is a challenge as the kidney is an architecturally and functionally complex organ at both a microscopic and macroscopic level. However, recent studies have reported the directed differentiation of iPSC to podocytes [45], tubular structures [46, 47] and self-organising renal organoids [48], providing a potential *in vitro* model system with which to validate novel genetic findings in GRD patients. This also provides the potential to better understand underlying pathology with the aim of developing new treatments.

Recent advances in genetic sequencing technology have resulted in remarkable improvements in the speed, throughput and cost of sequencing all, or part, of an individual’s genome. While it would appear feasible to apply these new technologies to kidney disease, a clinical protocol is required to guide patient identification and recruitment, ethical acquisition of material, and appropriate counselling whilst coupling this with NGS for the identification of novel mutations and iPSC validation to discover the genetic basis for rare genetic diseases. The need for patients to be informed of outcomes must also be addressed. We hypothesize that emerging high-throughput sequencing technologies will lead to the rapid identification of novel causative genes in GRD and propose a study to begin realising this potential within Australia.

## Methods

### Study aim

This study aims to discover the genetic basis for disease in a cohort of patients thought clinically to have a genetic disorder causing renal dysfunction or disease. The cohort will be selected based upon family history and phenotype strongly suggesting a genetic aetiology and in whom routine genetic testing is not available, not feasible or has not identified a mutation/s in currently known genes. Our hypothesis is that we will be able to identify the disease-causing mutations in a proportion of these participants.

### Study design

**Fig. 1:**
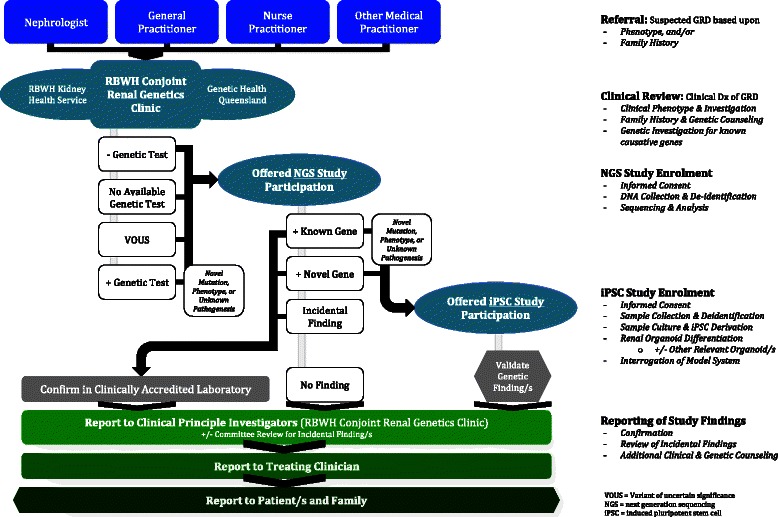
Study flow diagram

This is a translational study based at Royal Brisbane and Women’s Hospital (RBWH) and the University of Queensland (UQ) to discover, validate and explain new genetic causes for inherited kidney disease where investigation for known genes has been unsuccessful or is unavailable. Participants will be recruited from patients attending the RBWH Conjoint Renal Genetics and Inherited Kidney Disease Clinics, as part of standard clinical care (Fig. 1).

### Ethical considerations

The RBWH Human Research and Ethics Committee (approval HREC/14/QRBW/34), the UQ Medical Research Ethics Committee (approval 2014000453) and the Children’s Health Queensland Hospital and Health Service Human Research and Ethics Committee (approval HREC/15/QRCH/126), reviewed and approved the study.

### Target population

This study involves two groups of participants:

1. An affected individual whose family history and/or phenotype strongly suggests a genetic aetiology and in whom routine genetic testing:
  - Is not clinically available
  - Is not feasible given the suspected disorder has high genetic heterogeneity, or
  - Has already failed to arrive at a diagnosis
2. First degree and appropriate relatives of participants of group 1.

#### Patient identification and screening

Patients referred to the RBWH Kidney Health Service, RBWH Conjoint Renal Genetics Clinic and/or Genetic Health Queensland (RBWH) will be identified as potential research participants by their treating nephrologist and/or clinical geneticist.

Once a suitable individual from the target population is identified and consent obtained by the treating clinician, their first-degree relatives will be invited to participate in the study by consented individuals. The research team will then be available to discuss and provide full information about the study to the first-degree relatives and offer them the opportunity to participate, also by informed consent.

#### Patient/participant inclusion

In most cases inclusion in this study will require the participation of the patient and both parents. However, in some cases a combination of factors including the structure of the family pedigree, the suspected mode of inheritance, and information available regarding the specific disease may provide the required level of genetic information or require additional or alternate family members to be included in the study, and therefore warrant inclusion in the study. This will be determined on a case-by-case basis by the principal investigators.

#### Patient/participant exclusion

The criteria for participant exclusion include unwillingness to participate in the study and an insufficient number of direct relatives willing/able to participate in the study.

#### Patient/participant withdrawal

Participants may be withdrawn from the study in the following circumstances:

- *Withdrawal at participant request.* All participants have the right to withdraw from the study at any point in time. If a participant withdraws from the study the analysis of their DNA and genetic information will cease at that point. DNA samples and any sequenced genomic data generated will be destroyed.
- *Withdrawal due to diagnosis from standard care.* If a specific genetic diagnosis becomes available during the course of the study, the patient and the family will be withdrawn from the study. If the pathobiology of this diagnosis is not known or unclear, the participant may be offered the opportunity to continue with iPSC validation of the specific genetic diagnosis without participation in NGS.

### Participant identifiers

Upon recruitment, all participants will be assigned a unique identifier for the purposes of this study. A confidential database, maintained on a Queensland Health (QH) server, will link participant identifying information and their study identifier code. Study team members from The University of Queensland will receive all samples and case details anonymously, with the exception of the unique identifier. Only the clinical service will know the identity of the patient samples.

### DNA sample isolation

Peripheral blood is the preferred source of DNA for this study due to the relative high quality, reliability and yield of DNA compared to extractions from other tissues. Blood samples will be collected by a trained phlebotomist and DNA will be extracted by Pathology Queensland.

In some cases DNA samples will be required from a tissue other than blood in specific instances, for example, when validating the presence of mosaicism in a given individual. In these cases a buccal (cheek) cell swab or saliva sample will be taken by the treating nephrologist or clinical geneticist. Buccal samples will be taken using Qiagen Gentra Puregene Buccal Cell Kits (or equivalent) and saliva samples will be taken using Oragene saliva DNA collection kits.

### Summary of clinical data, family structure and family history

The treating clinician of each affected index participant will prepare a summary of the clinical features of the participant, the family structure and any relevant family history. This information is required to make informed evaluations of the genetic variant data derived from each family. This summary will not include any identifying information other than participant study identifier codes. This clinical summary will be provided to the research team when DNA samples are provided.

### DNA sequencing

Participant DNA samples will be sequenced using one or more next generation sequencing technologies. Participant samples may undergo whole genome sequencing, whole exome sequencing and/or targeted re-sequencing of restricted gene panels. The sequencing method will be customised depending on the clinical presentation of the condition and identity of any suspected candidate genes. It is expected that exome sequencing will be the predominant technique used to assess participant DNA due to the favourable equipoise between coverage of the genome and cost. Whole genomes will be sequenced to a minimum depth of 30× reads average coverage depth while exomes will be sequenced to a minimum depth such that >80 % of targeted regions are sequenced to a depth greater than 20× reads.

### Sequence analysis

DNA sequence data will be analysed from all participating members of a family in parallel. Comprehensive quality control metrics are derived for each sample to ensure the quality of the sequence data. All sequence reads are compared to the human reference sequence and used to identify variant positions where the participant DNA is different from the reference genome. Each variant position within a family is then evaluated based on its likelihood to contribute to the disease of the affected individual/s. This assessment will be augmented and assisted by the Variant Assessment Tool [49–51], an internal software tool that integrates family genetic variant segregation details with external referenced information sources such as the frequency of the allele in published control populations, the predicted impact of the variant on protein coding sequences and genes known to be associated with genetic disorders.

### Reporting of findings

At the completion of the analysis, the findings will be reported to the RBWH Kidney Health Service and RBWH Conjoint Renal Genetics Clinic principal investigators. These findings will then be presented to the treating clinician after patient re-identification using their unique study identifier. If candidate variants are identified that are believed to be involved in the patient’s renal disease, they will be validated using Sanger sequencing in an accredited clinical laboratory, if available. This information will then be provided to the treating clinician for discussion with their patient. If participants opted during the informed consent process not to receive their results, including any incidental findings discussed below, they will be asked to confirm this decision when the results of the research are available. Consent will be sought from the participant should relatives need to be approached regarding the research findings.

### Validation using participant-derived iPSC lines

Identification of a predicted disease causing mutation via any NGS approach requires validation, particularly if the mutation is novel for this phenotype. This may include identification of a similar mutation in another family, validation of disease segregation within the affected family or *in vitro* cell based analyses. This is critical in the evaluation of any NGS-predicted mutation. In a limited number of cases, and with patient consent, participant-derived primary fibroblasts have been isolated from affected individuals and clearly unaffected relatives for the purpose of derivation of induced pluripotent stem cells. Such iPSC can be used to generate specific kidney cell types [46–48]. Comparisons will be made between lines derived from the patient and the unaffected relative to partially control for variations due to the underlying genetic background. This requires the collection of fibroblasts (skin cells), which can be cultured as a primary cell line, from the proband and an unaffected relative. These cell lines will only be used for direct functional assessment of the participants’ genetic variants or where the specific inherited kidney disease’s pathobiology is unknown or incompletely understood. The cell lines will not be used for commercial purposes and will not be shared with third parties not directly involved in this study.

After obtaining specific participant consent, medical staff will obtain skin samples using a punch or shave biopsy, or during a surgical procedure should a participant be having surgery for an unrelated reason. Fibroblast culture will be undertaken according to established protocol [52]. Blood [53], urine [54] or buccal swab samples may also or alternatively be used to obtain a participant sample. Samples will be labelled with participant study unique identifier codes and transferred directly to the research laboratories for fibroblast and iPSC culture.

iPSC will be created from cultured patient cells with non-integrative reprogramming using Sendai virus transfection [55]. Subsequent differentiation to self-organising renal progenitors, structures and organoids will be undertaken [48]. A minimum of three clones from each participant (both affected patient and unaffected relative) will be assessed for disease phenotypes, to account for interclonal differences. Additional differentiation to other tissue types of relevance to the presentation of the participant will be performed as appropriate depending on the variant/s discovered and the participant phenotype revealed in the summary of clinical information accompanying the participant study unique identifier code. This is anticipated to include but not be limited to neural, retinal, hepatic, pulmonary and osseous tissues.

Participants may decline to provide skin, blood, urine or buccal swab samples; this will not affect their ability to participate in the genetic sequencing component of this study.

## Disclosure of results

Results will be returned to the treating clinician who will be able to re-identify the participant. The evaluation of the research finding and disclosure to the patient and their family will be the responsibility of the treating clinician. The research findings may include a result of uncertain significance or an uninformative result. Patients will be counselled on the potential clinical implications of the results, as is standard clinical care. Further testing in a clinically-accredited laboratory may need to take place should the result be required for clinical management.

The study may reveal results that are significant to family members. The research results will be provided to the patients in the clinic. They will be counseled, if relevant, on the need to inform their relatives of their results. These patients will be provided with a family letter they can send to their relatives. Relatives considered at risk can then obtain a referral to the RBWH Kidney Health Service, RBWH Conjoint Renal Genetics Clinic or Genetic Health Queensland (or their local nephrology or clinical genetics service) for counseling. Subsequent clinical genetic testing (cascade testing) may be arranged for these relatives.

Due to the nature of this analysis, any non-paternity/maternity present within a participating family will be detected during our analysis. In accordance with the Australian National Health and Medical Research Council guidelines (Medical Genetic Testing Information for Health Professionals 2010 [56]) non-paternity or non-maternity would be disclosed in only the most exceptional circumstances, as this may cause serious harm to individuals and families.

Results could potentially preclude participants from insurance such as life, income protection, and mortgage protection. It is an applicant’s responsibility to declare any known health information about themselves and their genetic relatives in insurance applications. This topic is covered in detail during the initial consent process.

### Incidental findings

One or more disease-associated genetic variants that are not related to the genetic condition being investigated may be identified in a participant. These are termed “incidental findings”. An example of this could be a gene mutation associated with an increased risk of a serious health problem, such as cancer. There is growing recognition of particular genes for which there is consensus that mutations in those genes may represent clinically significant incidental findings [57, 58]. This study has a very small chance of discovering an individual/family has a heritable disposition to an unrelated disorder (eg, cancer). The frequency of such actionable unintended findings is estimated to be less than 3 % [59, 60]. Participants will undergo a rigorous prospective consent process informing them of this, with the option of opting out from receiving such information in their study results.

If a genetic variant is discovered that the research team believes may be medically relevant for one or more of the participants, and the participant has consented to receive such incidental findings, it will be referred to an expert panel for review. The panel will consist of one clinical nephrology principal investigator (AM, HH or delegate), one person expert in next generation sequencing bioinformatics (RT, CS or delegate), one co­opted clinical geneticist (CP, MG, JM or delegate), and one co­opted medical specialist in the field to which the potential disease pertains (eg oncologist, gastroenterologist). The committee will consider the incidental finding and determine if (a) the finding confers a high risk of disease in the future; and (b) interventions such as surveillance are available to decrease the risk of morbidity/mortality. Should both criteria be fulfilled, the result will be presented to the participant if they had consented to learn of such information.

All participants who have previously declined to be informed of incidental findings will be asked again if this is still their wish, regardless of whether an incidental finding has been uncovered or not. All participants are informed of this at the time of initial consent.

## Discussion

Advances in NGS bring within reach the promise of identifying genetic changes associated with the majority of heritable diseases. The discovery of novel disease-causing genes and mutations for a specific condition results in substantial potential benefits to both the patient/family as well as other patients thought to have that condition (Table 1).

**Table 1:** Potential benefits of identifying novel disease-causing mutations in kidney disease

**•** Achieving a diagnosis
**•** Precise identification of the molecular defect for genotype/phenotype correlations
**•** Accurate genetic counselling and, where appropriate, subsequent prenatal testing
**•** Accurate cascade testing to identify risks to other relatives
**•** Enhanced gene lists for future diagnostic testing
**•** Elucidation of new genetic conditions/syndromes, improving diagnosis in further individuals
**•** Improved understanding of the molecular pathology, leading to the development of more effective therapies

Recently Simons et al. have used a combination of WES and WGS to study the genetic basis of a cohort of patients with leukodystrophy; a group of rare disorders characterized by dysfunction of the white matter of the brain. This approach has described several new diseases, including “hypomyelination with brain stem and spinal cord involvement and leg spasticity”, due to autosomal recessive mutations in the aspartyl-tRNA synthetase gene *DARS* [51], and “leukoencephalopathy hypomyelination with atrophy of the basal ganglia and cerebellum” due to de novo mutation in the gene *TUBB4A* [50]. This group has also recently identified the previously unknown genetic cause of Temple-Baraitser syndrome to be due to mutations in *KCNH1* [49]. Our study utilises and builds upon this clear skillset and expertise to discover and describe new genetic causes of GRD.

There are examples however where disease-causing genotypes are recalcitrant to or are unexplored by the application of NGS, such as in Autosomal Dominant Tubulointerstitial Disease (formerly known as Medullary Cystic Kidney Disease type 1 [61, 62] and type 2 [63–66]). Furthermore, whole exome or genome approaches, as used in gene discovery experimentation, are inaccurate in several relatively common and well-described diseases such as ADPKD [67], which instead require a tailored MPS approach [67, 68]. On the other hand, the power of diagnostic NGS approaches have been exemplified in Alport Syndrome [69], particularly with respect to identifying mosaicism [70] and unclear clinicopathological associations [71–73].

Whilst utilising such rapidly evolving genomic approaches in clinical practice offers significant potential benefits [74], it also creates potential complications. The perceived benefits of this technology have become a source of concern, particularly with regard to unintended or incidental findings [75–82]. This is further exacerbated by the variety of legal and regulatory paradigms within a globalised society [83–89]. The frequency of such actionable unintended findings in comparable studies to ours has been less than 3 % [59, 60]. If the criteria for “actionability” is liberalised this may be higher [90, 91], though still of potential personal and cost benefit [92].

One cannot presently pretend to have durable or all-encompassing answers to the conundrums posed by incidental genetic findings. Prudence and increased emphasis on informed consent and appropriate reporting may however provide a way forward [93–95]. A less definable answer is that local multidisciplinary teams provide broad opportunities to consider such challenges from a plurality of perspectives thus improving clinical translation, maximising benefits, minimising risk and providing durable clinical supports. Accordingly these have been actively integrated into the study we propose.

Functional validation of novel genetic discoveries in rare diseases poses significant challenges. iPSC technologies, as first described in 2006 [40], provide opportunities and unique strategies to address this. Patient-derived iPSC approaches to create, identify, describe and differentiate renal progenitors and self-organising organoids [48] provide the opportunity to undertake such validation via patient-derived iPSC “disease in a dish” studies. Further experience with other tissue phenotypes, as may be encountered in inherited syndromic multisystem disease, will be utilised. Specifically, Wolvetang et al. have successfully modelled human genetic disease (Ataxia-Telangiectasia and Down syndrome) using patient derived iPSC in studies that have further described the cellular and tissue pathobiology underpinning how genetic aetiology translates into complex clinical phenotypes [96, 97]. The application of CRISPR-Cas9 technology [98–100] to introduce or correct mutations in such cellular or organoid disease models [101–103] may also be required and employed. We propose that this coordinated approach to *in vitro* patient-derived iPSC study will enable a greater ability to characterize rare GRD at a cellular level and presents a logical pathway and opportunity for functional validation of genetic variants discovered.

In summary, we describe a collaborative research approach to the elucidation of the underlying cause of previously refractory forms of GRD. The protocol requires integration between clinicians and scientists and the application of state-of-the-art genetic and stem cell technologies within diagnostic proximity of the patient in order to reduce the number of cases of unknown genetic causation. There remain significant challenges to delivering new knowledge to the clinician and patient, including the bioinformatics challenges of identifying a causative mutation out of the >60,000 variations likely to be detected in any individual, and the technical obstacles to the functional analysis of iPSC-derived organoids. If adopted more broadly, this approach can potentially translate into optimised diagnostic, therapeutic and clinical outcomes for affected patients.

## Abbreviations

GRD: Genetic renal disease
NGS: Next generation sequencing
MPS: Massively parallel sequencing
WES: Whole exome sequencing
WGS: Whole genome sequencing
iPSC: Induced pluripotent stem cell
RBWH: Royal Brisbane and Women’s Hospital
UQ: The University of Queensland
VOUS: Variant of uncertain significance
